# Controversies surrounding human papilloma virus infection, head & neck vs oral cancer, implications for prophylaxis and treatment

**DOI:** 10.1186/1758-3284-1-8

**Published:** 2009-03-30

**Authors:** Giuseppina Campisi, Lucia Giovannelli

**Affiliations:** 1 Settore di Medicina Orale, Dip. di Scienze Stomatologiche, Università Palermo, Via del Vespro 129-90127, Palermo, Italy; 2 Servizio di Virologia, Dip. di Scienze per la Promozione della Salute, Università Palermo, Via del Vespro 129-90127, Palermo, Italy

## Abstract

Head & Neck Cancer (HNC) represents the sixth most common malignancy worldwide and it is historically linked to well-known behavioural risk factors, *i.e.*, tobacco smoking and/or the alcohol consumption. Recently, substantial evidence has been mounting that Human Papillomavirus (HPV) infection is playing an increasing important role in oral cancer. Because of the attention and clamor surrounding oral HPV infection and related cancers, as well as the use of HPV prophylactic vaccines, in this invited perspective the authors raise some questions and review some controversial issues on HPV infection and its role in HNC, with a particular focus on oral squamous cell carcinoma.

The problematic definition and classification of HNC will be discussed, together with the characteristics of oral infection with oncogenic HPV types, the frequency of HPV DNA detection in HNC, the location of HPV-related tumours, the severity and prognosis of HPV-positive HNC, the diagnosis of oral HPV infection, common routes of oral infection and the likelihood of oro-genital HPV transmission, the prevention of HPV infection and novel therapeutic approaches.

## Background

Taking into consideration the attention and clamor surrounding Human Papilloma Virus (HPV) infection and related cancers, HPV vaccine controversies, as well as the recent Nobel Prize awarded to Prof. zur Hausen for his research on HPV and cervical cancer, authors believe it opportune, in this invited perspective, to raise various questions and review some of the most controversial issues related to HPV and its role in Head & Neck Cancer (**HNC**) and particularly in oral squamous cell carcinoma (**OSCC**), as well as the diagnosis of oral HPV infection and its oro-genital transmission.

**HNC **represents the sixth most common malignancy worldwide [1], and it is historically linked to several behavioural risk factors (*i.e.*, tobacco smoking and/or the consumption of alcohol). However, this term defines a heterogeneous group of malignant tumours, involving several different several sub-sites. The majority of HN malignancies are squamous cell carcinomas (**SCC**) and they originate from the epithelium which lines the upper aero digestive tract, *i.e.*, the oral cavity, the pharynx and the larynx. Arising in a multistep process resulting from the gathering of genetic and epigenetic defects and clonal spreading out of given cell populations [2], HN malignancies are known as HNSCC.

**HPV**s are epitheliotropic oncogenic DNA viruses with more than 120 identified genotypes: the so-called high-risk (HR) HPV, like HPV 16 and 18, have been definitively recognised as being strongly associated with ano-genital (cervical) cancers. In patients with high-grade neoplasia and cancer, HR HPV DNA can usually be found integrated into host DNA. The oncogenic potential of HR HPV is attributable to its ability to insert specific DNA fragments (early genes E6 and E7) into the host cellular genome. As a result of this integration, various key functions of tumour suppressor factors (the p53 and pRb pathways) are abrogated, leading to defects in apoptosis, DNA repair mechanisms, cell cycle regulation and, finally, to cellular immortalization, thus inducing and maintaining the malignant phenotype.

The HPV involvement in HN carcinogenesis was first proposed in 1983 by Syrjanen et al. [3] and then supported by several other authors on the basis of the following evidence: 1) the well-assessed broad epitheliotropism of HPV; 2) the morphological similarities between oropharyngeal and genital epithelia [4]; 3) the ability of immortalizing human oral keratinocytes *in vitro *[5]; and 4) the strongly established etiological role of HR HPV in cervical SCC [6,7].

Whilst occurring in a lower percentage than in cervical mucosa, HR HPV E6/E7 transcripts and/or viral integration have also been detected in HNC [8] and, it has, therefore, been suggested that HR HPV (mostly HPV 16, 18, 33) are involved in the viral-dependent inactivation of p53 and Rb. This occurrence also justifies the increased incidence of HNC and the onset of the tumour in younger people [9], whether the most common risk factors are present or not. As well as being associated with genital and oral mucosa diseases, HPV infection has also been reported in cases of nasal inverting papilloma, which is usually a benign tumour but is associated with squamous cell carcinoma (SCC) in about 10% of cases. Previous analyses have identified HPV-16 DNA in 32% of inverted papillomas and 58% papillomas associated with carcinomas [10]. More recently, an active role in the malignant lesion has been suggested for HPV on the basis of the presence of the HPV oncogene E6 and E7 transcripts, indicating the integration of the viral genome [11]. However, further analysis is required to confirm that HPV is not only a bystander agent.

In addition to these logical statements, it is timely to raise the following questions and shed light on these controversies:

### What is the frequency of HR HPV in HNC?

A very wide range of viral prevalence (0%–100%), in addition to the presence of HR HPV in oral normal mucosa [12,13], has been reported in the literature, even if with limited information regarding the natural history of oral HPV infection. Specifically relating to HPV 16 and 18, Kreimer et al [14] identified these genotypes in 16.0% and 3.9% respectively of 2,642 HNC reviewed in a recent systematic review of the literature; they calculated an overall prevalence of HPV in 25% of HNC vs 35.6% in oro-pharyngeal cancer and 23.5% in OSCC. Excluding any ethno-geographical bias among the patient groups examined, this wide range depends mainly on two variables: *i) *the site of the mucosa examined; and *ii) *technical issues (or the HPV molecular assay employed).

### What is the precise location of the HPV-related tumour?

A great deal of confusion is created by the use of the generic term *HNC*, *HNSCC *or *oral cancer *in place of the specific *OSCC*, *oro-pharyngeal *or *laryngeal carcinoma*. This issue plays a critical role since specific epithelial areas of the upper aero-digestive tract (such as the squamous-columnar junction at the level of the tonsillar crypts and the glottides) display greatest susceptibility to HPV due to the easy exposure of the basal cells, and this is also the case with the meta-plastic epithelial area in the cervix. Hence, it is critical to group HNSCC together as a single entity with the consequent difficulty of comparing the data. Recent epidemiological and molecular data have indicated the involvement of HR HPV in a given subset of HNSCC [14-19] (i.e. oro-pharyngeal and Waldeyer's tonsillar ring SCC) have been found to be significantly related to HR HPV(especially HPV 16/18). HPV 16 was very recently identified in tonsil-related cancers (palatine tonsil and at the base of the tongue) in oral exfoliated cells together with serum antibodies against HPV 16 [20], in non-smokers and non-drinkers, and oro-pharyngeal SCC patients [21]. Comparing these data, no clear results for OSCC and HR HPV have so far emerged. Various studies report a significant association between HPV 16 and to a lesser extent HPV 18 with OSCC [13,22-25] with respect to the frequency of HPV in normal oral mucosa. In a recent meta-analysis [26] of studies (1988 – 2007) on HPV in HNSCC vs OSCC biopsies, we found that the pooled prevalence of HPV DNA in the overall samples was 34.5%, while it was 38.1% in OSCC and 24.1% in the non site-specific HNSCC group. Unfortunately, it was confirmed that only a few studies had observed a correct distinction between cancers at oral and oro-pharyngeal sites, as recommended by the American Joint Committee on Cancer. Finally, the misclassification of some HPV-positive oro-pharyngeal cancers, such as OSCC, could partly explain the HPV-positivity of some "oral" cancers, thus even diminishing the real impact of HR HPV on oro-pharyngeal tumour onset [14,17].

### What type of infection is a prerequisite for carcinogenesis?

As gleaned from the field of gynaecology, a persistent viral status represents a necessary although not sufficient basis for HPV-related lesions [7,27-31]: persistent infection with a specific HR-HPV type must be maintained for at least 2 years and usually copies of the virus are found in great numbers in cervical mucosa. However, the virus is rarely found in oral mucosa, probably due to saliva clearance. Potentially, the probability that tonsillar crypts and the glottides will constantly harbour virions is greater than in other mucosal oral districts, and they will be detected with greater difficultly.

### Is there any protein expression/overexpression which is strongly associated with HR HPV in HNC?

Our first thought regards p16 and indeed it has been recently proposed as a surrogate marker of HPV DNA infection for oropharyngeal cancers [32]. The results for OSCC are less definitive [33-37], although some recent studies performed by real-time PCR [38], tissue microarray [39] and on a mouse model [40] have revealed the ability to identify (due to an over-expression of p16) those OSCC in which HPV infection was biologically significant.

### Does HPV positive oral cancer present a better prognosis and a different radio-chemo-sensitivity?

With reference to recent clinical data in the literature, HPV has been found to be the most significant positive prognostic factor in patients with oro-pharyngeal tumors, with a 60–80% reduction in the risk of death [41]. The favourable outcome of HPV-induced oropharyngeal cancers might be attributable to the absence of field cancerization or enhanced radiation sensitivity [42]. Taking this into consideration, the diagnosis of HPV infection should be determined in all oropharyngeal cancers, considering its presence as a key factor in the decision-making process of treatment [43-45]. However, we are unable to make the same suggestion for the OSCC. Furthermore, HPV 16 has been positively associated with a response to chemo-radiation in oro-pharyngeal cancer and with overall and disease-specific survival [46], whereas an HPV 16 positive cell line of HNSCC was recently found to be extremely cisplatinum-resistant [47].

### How difficult is it to make a diagnosis of oral HPV infection?

When performing the molecular detection of HPV DNA from oral samples, attention should be focused on the low productivity of oral HPV infections. Indeed, studies based on polymerase chain reaction (PCR) assay have shown that, when compared to those from HPV-positive cervical specimens, HPV-DNA positive oral samples from both normal oral mucosa [13,48] and cancerous lesions [49] produce weaker PCR products. Based on such data, it is then essential that, in clinical and research HPV testing, all procedures employed are highly sensitive, specific and reliable. In addition, the aim of enhancing the standardization of the approach, in terms of the type of oral specimen examined, sampling method applied and HPV molecular assay employed, should be pursued. As far as the oral sample to be examined is concerned, both tissue samples and superficial cells have been evaluated in literature [48,50,51]. Biopsy tissue samples can be either frozen at -80°C and then minced without thawing for DNA analysis or they can be formalin-fixed and paraffin-embedded. Oral mucosa exfoliated cells can be obtained either by oral brushing or rinse, and advantages and disadvantages have been reported for each method. Tissue samples allow for the histo-pathological examination of the biopsies used for the HPV test, as well as permitting the *in situ *hybridization and localization of HPV DNA in infected cells. However, the highest rate of HPV DNA is reported in DNA from frozen tissue, but the value is much lower for paraffin-embedded tissue samples [48]. Oral scrapes or rinse samples, with their greater surface area of mucosa than with biopsies, are less invasive, and HR HPV detection in oral exfoliated cells is a reliable biomarker of an HPV-related HN cancer risk. A drawback is that not all patients who have HR-HPV types in oral exfoliated cells are detected with HPV DNA in the primary tumour [50]. However, if HPV testing of oral exfoliated cells has been selected, the use of oral rinses would appear more efficient, in terms of cell yield and DNA-containing nucleated cells, compared with the superficial brushing/scraping of oral mucosa (by using a cotton swab, wooden spatula or a cytobrush) [51,52]. Of the several mouthwashes tested in the literature, commercial mouthwashes (*e.g.*, Cepacol^®^, Listermint^®^) would seem more efficient than sucrose, glucose and saline, in terms of DNA yield, quality and stability [53]. As far as HPV detection methods are concerned, it has been reported that methods such as Southern blotting and *in situ *hybridization should be avoided as they lead to lower HPV rates, compared with those obtained by using PCR assays [54,55].

As is the case with the most validated sampling procedures, it should be kept in mind that there are several other variables which may affect the efficiency and reliability of HPV detection in oral samples. For instance, the method of DNA purification also has a potentially large impact upon the ability to detect HPV DNA by PCR amplification [56]. Additionally, considerable differences exist regarding the use of different PCR primers. In the majority of the PCR-based HPV detection systems, a broad spectrum of HPV types is amplified by consensus primers, followed by detection with type-specific probes. The consensus primers may be either degenerate (as in the MY09/11 systems), or they may contain mismatches (as in the GP5+/6+ system), or they may contain inosine residues at ambiguous base positions (as in the SPF primers), or sets of overlapping primers (as in the PGMY primers). These methods have different analytical and clinical characteristics [57], and every method has its strengths and weakness. Although difficult to achieve, standardization and the use of validated procedures for HPV DNA is paramount in assisting physicians to provide more effective treatment and more efficient screening for patients.

### What is the most common route of oral HPV transmission?

Even though modes of HPV transmission in the head and neck mucosal districts have not been fully resolved, theories have proposed multiple pathways for HPV transmission, including perinatal transmission, auto-infection from oral-genital contact by hand and sexual transmission by oral-genital contact. The perinatal transmission of HPV to neonates at birth has been detected in several studies which have demonstrated that recurrent respiratory papillomatosis is associated with the perinatal transmission of HPV. While the possibility of auto-infection among women with cervical HPV infection is still a matter of debate [52,58-60], oral sex, including fellatio and cunnilingus, has being hypothesized as being the main mode of transit for oral HPV infection. Recently, HPV was detected more commonly in biopsy specimens from cancer patients with more than one sexual partner and from those who practiced oral sex than in biopsy specimens from those who did not engage in oral sex, thereby confirming the possibility of oral transmission [61]. However, mouth-to-mouth transmission, for example through kissing, still remains possible and it should not be excluded as a route of oral HPV transmission. Since the onset of the HIV epidemic, an increase in oral sex among teenagers and young adults has been observed, probably because this is thought to represent a form of safe sex. However, oral sex is not free of risk and it can result in HPV-related cancer. Public education is of paramount importance: there is a need to disseminate these findings and to place them in context. Even though the transmission of genital HPV infection primarily occurs via sexual contact, HPV-related diseases such as condyloma acuminata and laryngeal papillomas occur in neonates and children [62], suggesting additional modes of viral transmission such as perinatal infection during the passage through an infected birth canal [63] and *in utero*, as a transplacental or ascending infection [64,65]. Caesarean deliveries do not protect neonates against HPV [66] and HPV infection may be associated with adverse pregnancy outcomes, including spontaneous preterm delivery [67].

### Is HPV prevention possible and useful?

Although the use of condoms has proven efficient in the prevention of most sexually-transmitted infections, the effectiveness for prevention against HPV infection is not as clear, and a significant variability (from 0% to 80%) in the condom protection of HPV infection has been reported [68]. Alternatively, HPV infection could be prevented by the use of type-specific vaccines. In recent years, two HPV vaccines have been developed and are they available for primary vaccination in the European Union: a vaccine against HPV-16 and HPV-18 (Cervarix, produced by GlaxoSmithKline), which is administered in three doses (time 0, 1, and 6 months), and a quadrivalent vaccine against HPV-16, HPV-18, HPV-6 and HPV-11 (produced by Merck and distributed in Europe by Sanofi Pasteur MSD as Gardasil), which is administered in three doses (time 0, 2, and 6 months). The impressive range of protection, ranging from 86% to 100%, of the HPV vaccines has been recently reported [69,70]. The bivalent vaccine is advised for a reduction of precancerous cervical lesions and cancer incidence. In addition to cervical cancer prevention, the quadrivalent vaccine is advised for a reduction in genital condylomas. It is thought that these HPV vaccines could have broader implications, also for other HPV-related cancer in both women and men, thereby preventing oral as well as genital infections. This has prompted many researchers to advocate vaccinating boys as well as girls, with the bivalent vaccine to prevent HNC and by the tetravalent vaccine to additionally prevent oral condilomatosis. Thus, the question is "*Can oropharyngeal cancer now be placed in the category of virally-mediated cancers?"*, like, for instance, HPV-related cervical, anal, vulvar, and penile cancers, or the Epstein Barr virus-associated nasopharyngeal cancers. Whilst it is premature to imagine an HPV vaccine which could protect women and men against HNC, the possibility that various oral, oro-pharyngeal, and laryngeal cancers might be prevented by HPV vaccination is certainly a hope, and this would bring forth significant implications from a public health perspective.

### Beyond the vaccines, the potentiality of gene therapy

HPV vaccines should eventually reduce the impact of these viruses on human health. However, vaccines may not be useful for the treatment of existing disease, and it is necessary to develop effective therapies targeting those individuals who are already infected or are currently excluded from the first phase of a prophylactic vaccination program. Given the strong relationship between the expression of HPV E6 and E7 and cervical cancer carcinogenesis, many approaches have been directed against these oncogenes, for example, gene therapy for HPV-positive cervical cancers [71]. The different methods also include the treatment of cervical cancer with E6 short interfering RNA, the use of antisense RNA to E7 and E6 genes either alone or together, and the use of mutated E2 protein that acts as a cancer cell-specific inducer of apoptosis [72-74]. In *in vitro *studies on antisense HPV RNA transcripts, the transcripts of the E6 and E7 genes of HPV type 16 were introduced (via a recombinant adenoviral vector, *e.g.*, Ad5CMV-HPV 16 AS) into human cervical cancer cells harbouring HPV 16; the effects of expression of these genes on cell and tumour growth were then analysed. It was found that, when E6 and E7 protein expression is suppressed, p53 and Rb protein expression increase, the Ad5CMV-HPV 16 AS-infected cells undergo apoptosis, and cell growth and tumourigenicity are greatly suppressed. However, even though gene therapy may prove to be beneficial in the treatment of cervical cancer and other HPV-induced diseases, a further understanding of the viral life cycle and the mechanisms underlying HPV-induced oncogenesis is necessary before this method could be employed for clinical use in humans.

## Competing interests

The authors declare that they have no competing interests.

## Authors' contributions

All authors read and approved the final manuscript.
